# Onset-Duration Matching of Acoustic Stimuli Revisited: Conventional Arithmetic vs. Proposed Geometric Measures of Accuracy and Precision

**DOI:** 10.3389/fpsyg.2016.02013

**Published:** 2017-01-06

**Authors:** Björn Friedrich, Peter Heil

**Affiliations:** Systems Physiology of Learning, Leibniz Institute for NeurobiologyMagdeburg, Germany

**Keywords:** psychophysics, method of adjustment, positive-real numbers, log-ratio metric, convexity bias, illusions, constant error, Weber fraction

## Abstract

Onsets of acoustic stimuli are salient transients and are relevant in humans for the perception of music and speech. Previous studies of onset-duration discrimination and matching focused on whether onsets are perceived categorically. In this study, we address two issues. First, we revisit onset-duration matching and measure, for 79 conditions, how accurately and precisely human listeners can adjust the onset duration of a comparison stimulus to subjectively match that of a standard stimulus. Second, we explore measures for quantifying performance in this and other matching tasks. The conventional measures of accuracy and precision are defined by arithmetic descriptive statistics and the Euclidean distance function on the real numbers. We propose novel measures based on geometric descriptive statistics and the log-ratio distance function, the Euclidean distance function on the positive-real numbers. Only these properly account for the fact that the magnitude of onset durations, like the magnitudes of most physical quantities, can attain only positive real values. The conventional (arithmetic) measures possess a convexity bias that yields errors that grow with the width of the distribution of matches. This convexity bias leads to misrepresentations of the constant error and could even imply the existence of perceptual illusions where none exist. This is not so for the proposed (geometric) measures. We collected up to 68 matches from a given listener for each condition (about 34,000 matches in total) and examined inter-listener variability and the effects of onset duration, plateau duration, sound level, carrier, and restriction of the range of adjustable comparison stimuli on measures of accuracy and precision. Results obtained with the conventional measures generally agree with those reported in the literature. The variance across listeners is highly heterogeneous for the conventional measures but is homogeneous for the proposed measures. Furthermore, the proposed measures show that listeners tend to under- rather than to overestimate the onset duration of the comparison stimuli. They further reveal effects of the stimulus carrier on accuracy and precision which are missed by the conventional measures. Our results have broad implications for psychophysical studies that use arithmetic measures to quantify performance when geometric measures should instead be used.

## 1. Introduction

Onsets are salient features of the temporal envelopes of acoustic stimuli, with perceptual and behavioral relevance in a variety of species from insects to humans. For example, the onset duration, also called rise time or attack time, of sound pulses modeling songs of female grasshoppers determines whether a male turns toward the sound source (von Helversen, 1993). Similarly, phonotactic responses of female tree frogs to synthetic male mating calls depend on the onset duration of the calls (Gerhardt and Schul, 1999). In humans, characteristics of the onset influence the timbre of music and speech sounds (e.g., McAdams et al., 1995; Lakatos, 2000; Halpern et al., 2004). For instance, the auditory perception of a trumpet can be transformed into that of a violin, and vice versa, by adequately manipulating the properties of the sound signal during the initial 50 ms or so (Grey and Gordon, 1978). That the onset is crucial for the identification of an instrument is also supported by experiments showing that when instrumental sounds with asymmetrical onsets and offsets are played backward—a manipulation that does not alter the long-term spectrum—human listeners often fail to recognize the instrument (Paquette and Peretz, 1997). In speech, studies of general grouping principles of speech components imply that the onset of a sound's envelope is more important than what follows (Darwin, 1981).

Cutting and Rosner (1974, 1976) and Cutting (1982) suggested that non-speech sounds varying along an onset-duration continuum are perceived categorically and that onset duration can therefore cue categorical perception, such as that of music and speech sounds. Rosen and Howell (1981) showed, however, that the peak in the discrimination performance observed by Cutting and Rosner (1974, 1976) was due entirely to the stimuli not having the intended onset durations. For stimuli having the intended onset durations, discrimination performance decreased monotonically with increasing onset duration (Rosen and Howell, 1981, 1983). Other investigators also questioned the categorical perception of the onset-duration continuum and suggested that the ability to discriminate or match onset durations rather follows Weber's law (Tenney, 1962; Pollack, 1963; van Heuven and van den Broecke, 1979; Hary and Massaro, 1982; van den Broecke and van Heuven, 1983; Kewley-Port and Pisoni, 1984; Smurzyński, 1985; Smurzyński and Houtsma, 1989), which states that the Weber fraction is constant.

**Figure 1:**
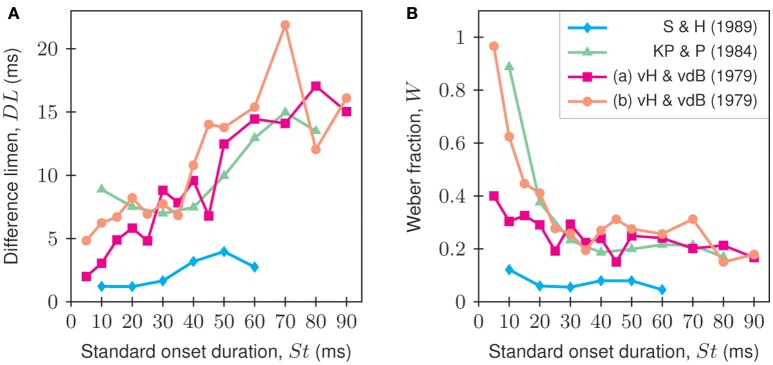
**Difference limens (A)** and corresponding Weber fractions **(B)** as functions of standard onset duration from four studies in the literature. The Weber fractions were computed by dividing the difference limens by the standard. Blue: data from Smurzyński and Houtsma (1989) (carrier: 1000 Hz; total duration: 256 ms; decay duration: 40 ms; level: 70 dB SPL; linear rise and decay; procedure: adaptive 2-IFC; listeners: *n* = 4; average: arithmetic mean). Green: data from Kewley-Port and Pisoni (1984) (carrier: sawtooth; total duration: 1000 ms; decay duration: total duration minus onset duration; level: 82 dB SPL; linear rise and decay; procedure: adaptive 2-IFC; listeners: *n* = 4; average: median). Magenta and orange: data from van Heuven and van den Broecke (1979) (carrier: 1000 Hz (a) or white noise (b); plateau duration: 400 ms; decay duration: 50 ms; level: 60 dB; linear rise and decay; procedure: method of adjustment; listeners: *n* = 8; average: arithmetic mean).

Reported Weber fractions vary widely among studies—between 6 and 8% (Smurzyński and Houtsma, 1989) and 100% or even more (Pollack, 1963). However, Weber fractions are approximately constant over only a limited range of onset durations. Below this range, they consistently increase with decreasing onset duration (Tenney, 1962; Pollack, 1963; van Heuven and van den Broecke, 1979; van den Broecke and van Heuven, 1983; Kewley-Port and Pisoni, 1984). Moreover, Weber fractions may decrease with training (Smurzyński and Houtsma, 1989), and they depend on stimulus level. Kalmylova and Shakhshaev (1988) showed, for a single onset duration of 15 ms, a decrease in the Weber fraction with increasing sound level. Figure 1 shows difference limens and corresponding Weber fractions reported in four studies. The small Weber fractions from Smurzyński and Houtsma (1989), who used tone carriers and a sound level of 70 dB SPL, are in line with the finding of Kalmylova and Shakhshaev (1988) that Weber fractions are smallest (onset-duration discrimination is best) at high sound levels. Weber fractions reported by Kewley-Port and Pisoni (1984), who used broadband sawtooth carriers, are larger and increase more rapidly with decreasing onset duration below about 30 ms than those observed by Smurzyński and Houtsma (1989), even though the former used an even higher sound level (82 dB SPL). It therefore seems that the type of carrier might also affect the Weber fraction. An influence of the carrier can also be inferred from the study of van Heuven and van den Broecke (1979), who obtained somewhat smaller Weber fractions for stimuli with tone than with broadband noise carriers of the same sound level (60 dB), though the data are noisy.

Van Heuven and van den Broecke (1979) and van den Broecke and van Heuven (1983) used the method of adjustment, a fundamental and classical psychophysical procedure devised by Fechner (1860). In this method, listeners adjust a variable comparison stimulus (Co) until it subjectively equals a fixed standard stimulus (St) serving as a reference. Ideally, this is repeated many times, so that for each St there is a distribution of comparison stimuli that the listener accepted as a match. The center of this distribution is the point of subjective equality (PSE), and the signed distance between the PSE and the St is the constant error (CE), also referred to as the systematic error. The more accurate the adjustments are, the smaller the magnitude of the CE. The sign indicates the direction of the CE. A measure of the width of the distribution is used to define the difference limen (DL), conceived of as the minimum amount the stimulus must change to produce a just-noticeable difference (JND) in the sensation. The more precise the adjustments are, the smaller the DL. Relative precision is often quantified by the Weber fraction (W), commonly defined as the ratio of the DL to the St, *W* = *DL*/*St*. The listeners in the study of van Heuven and van den Broecke (1979) made only two matches per condition, and difference limens and Weber fractions were calculated from matches pooled across all listeners. Moreover, constant errors were not reported, although, as we will show in the Results section, constant errors and Weber fractions may interact.

We therefore believe that a thorough reexamination of onset-duration matching with respect to stimulus factors affecting the accuracy and precision of adjustments, as well as with respect to inter-listener variability, is in order. To reexamine onset-duration matching, we performed three experiments with up to ten listeners each. In Experiment 1, we explored the dependence of accuracy and precision on onset duration (varying from 0.5 to 64 ms), sound level (varying over a 24-dB range of low sensation levels), and carrier (tone and noise). In Experiment 2, we examined whether accuracy and precision depend on plateau duration, which covaried with onset duration in Experiment 1. In Experiment 3, we examined how restricting the range of comparison stimuli affects accuracy and precision. In all experiments, each listener performed many matches for each condition, allowing us to characterize the distributions of matches, to derive reliable estimates from each listener and condition, and to explore inter-listener variability.

The second major focus of this study concerns the way in which accuracy and precision should be quantified. We believe that the statistical measures used to quantify accuracy and precision in tasks using the method of adjustment deserve reexamination. Matches to a given standard are typically conceived of as realizations of a random variable, *X*_*St*_, with distributions over the Euclidean standard vector space ℝ. In this space, vector addition and scalar multiplication are defined as usual addition and multiplication, respectively, and the Euclidean distance is given by the function

(1) $${\text{dist}}_{\mathbb{R}}\;(x,y)=|x-y|.$$

For convenience, we hereafter refer to this additive structure simply as ℝ. In line with the additive structure of ℝ, the textbook definitions (see e.g., Guilford, 1954; Gescheider, 1997; Ramajanickam, 2002; Mapp and Ono, 2006) of the four psychophysical measures, PSE, CE, DL, and W, are computed from *X*_*St*_ using arithmetic descriptive statistics such as the arithmetic mean AM(*X*_*St*_), arithmetic variance AV(*X*_*St*_), and arithmetic standard deviation AS(*X*_*St*_). These conventional (arithmetic) psychophysical measures are summarized in Table 1.

**Table 1:** **Conventional and proposed psychophysical measures, computed from the matches to a given standard (***St***), realized as a random variable ***X***_***S**t*_**.

**Psychophysical measure**	**Conventional (arithmetic)**	**Proposed (geometric)**
Point of subjective equality	*PSE*_A_ = AM(*X*_*St*_)	*PSE*_G_ = GM(*X*_*St*_)
Absolute constant error	abs. *CE*_A_ = *PSE*_A_−*St*	—
Relative constant error^†^	$CE_{\text{A}}=\frac{PSE_{\text{A}}-St}{St}$	$CE_{\text{G}}=ln\;\left(\frac{PSE_{\text{G}}}{St}\right)$
Difference limen	*DL*_A_ = AS(*X*_*St*_)	*DL*_G_ = GS(*X*_*St*_)
Weber fraction	$W_{\text{A}}=\frac{DL_{\text{A}}}{St}$	*W*_G_ = ln (*DL*_G_)

*The conventional measures are based on arithmetic descriptive statistics (subscript “A”) and the distance function dist_ℝ_ of the Euclidean vector space ℝ. Our proposed measures are based on geometric descriptive statistics (subscript “G”) and the log-ratio distance function dist_ℝ>0_ of the Euclidean vector space ℝ_>0_. The measures are derived in the section*.

† *Note that we write CE_A_ instead of rel.CE_A_*.

However, the magnitudes of most fundamental (e.g., mass, length, duration) and many derived (e.g., area, volume, density, pressure, frequency) physical quantities can attain only positive real values (BIPM, 2006; Gupta, 2010). Even though the set of positive-real numbers, ℝ_>0_, is a subset of the set of real numbers, ℝ, it is not a subspace in terms of the vector space structure of ℝ. Instead, ℝ_>0_ has its own Euclidean vector space structure in which multiplication and exponentiation naturally take on the role of vector addition and scalar multiplication. Again, for convenience, we hereafter refer to this multiplicative structure simply as ℝ_>0_. The Euclidean distance, the metric induced by the standard-scalar-product norm, is given by the log-ratio distance function

(2) $$\begin{array}{ll} {\text{dist}}_{{\mathbb{R}}_{>0}}(x,y) & =|\ln\left(\frac{x}{y})\right|=|\ln(x)-\ln(y)|. \end{array}$$

This metric measures relative rather than absolute differences between stimuli and has a number of other interesting properties (e.g., Graff, 2014). Consistent with the structure of ℝ_>0_ are the geometric mean GM(*X*_*St*_), geometric variance GV(*X*_*St*_), and geometric standard deviation GS(*X*_*St*_) of the random variable *X*_*St*_ over ℝ_>0_. Formulae to calculate the arithmetic and the geometric statistical measures are summarized in Table 1 of the Supplementary Material. The most important operations and properties of the two Euclidean vector spaces ℝ and ℝ_>0_ are compared in Table 2 of the Supplementary Material.

It is important to realize that arithmetic descriptive statistics possess a convexity bias when computed from random variables over the multiplicative Euclidean vector space of positive-real numbers, and that this convexity bias is present in the conventional psychophysical measures. This can be demonstrated in a straightforward manner with the following Gedanken experiment. Let *X*_*St*_ follow a log-normal distribution, also called a multiplicative normal distribution (Limpert et al., 2001; Limpert and Stahel, 2011)—the canonical probability distribution with support on the Euclidean vector space ℝ_>0_. We hereafter use the terms *multiplicative normal* and *additive normal* for the log-normal and the normal distributions, respectively. Let us further assume the special case that the subject is perfectly accurate, such that the center of the multiplicative normal distribution is exactly equal to the standard, i.e., GM(*X*_*St*_) = *St*. The logarithm of *X*_*St*_ then follows an additive normal distribution (N) with mean μ = ln(*St*) and variance σ^2^, such that $\ln\left(X_{St}\right)\sim N\left(\mu ,\sigma^{2}\right)$. Using known relationships between statistical descriptive measures and the parameters of a multiplicative normal distribution (see Table 3 of the Supplementary Material), one can reformulate the famous inequality AM(*X*_*St*_) ≥ GM(*X*_*St*_), a special case of Jensen's inequality ln(AM(*X*_*St*_)) ≥ AM(ln(*X*_*St*_)), to obtain an equality with a known bias factor, namely the square root of the geometric variance:

(3) $$\begin{array}{ll} \text{AM}(X_{St}) & =\text{GM}(X_{St})\;\cdot \;\sqrt{\text{GV}(X_{St})}\;. \end{array}$$

**Figure 2:**
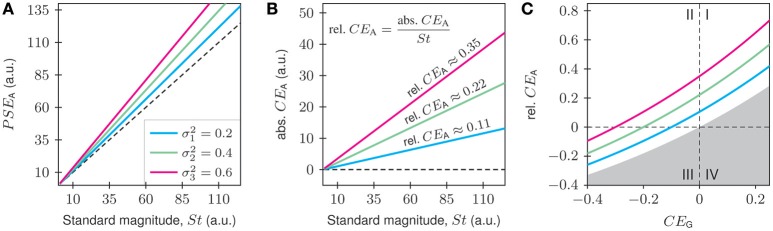
**Theoretical relationships between conventional psychophysical measures and the standard in the case of multiplicative-normally distributed matches, shown for three different geometric variances, e^**σ**^**2**^^ (see legend in A)**. In **(A,B)**, the geometric means are always equal to *St* (perfect accuracy), whereas in **(C)**, they range from below to above *St*. **(A)** The slopes of the *PSE*_A_-vs.-*St* functions exceed 1, the more so the greater the geometric variance. **(B)** The conventional analysis suggests absolute constant errors which are positive (abs. *CE*_A_ > 0; black dotted line denotes zero units) and increase linearly with *St*. The corresponding relative constant errors are independent of *St*, but increase with increasing geometric variance. They are given by the (dimensionless) slopes of the lines relating the absolute *CE*_A_ to *St*, that is, $\text{rel}.CE_{\text{A}}=\frac{\text{abs}.CE_{\text{A}}}{St}$. Our proposed measure of accuracy, the geometric constant error $CE_{\text{G}}=\text{ln}\;\left(\frac{PSE_{\text{G}}}{St}\right)$ derived in the Results section, is 0 (not shown here). **(C)** If the subject is not perfectly accurate, *CE*_G_ ≠ 0. When *CE*_G_ ≥ 0, then *CE*_A_ > 0 (quadrant I). When *CE*_G_ < 0, then *CE*_A_ can be negative (quadrant III) or positive (quadrant II), depending on the magnitude of *CE*_G_ and on the geometric variance. The gray region represents impossible combinations of rel. *CE*_A_ and *CE*_G_.

The name convexity bias originates from the fact that the logarithm is a negatively convex function. The impact of the convexity bias on the conventionally defined PSE and CE is illustrated in Figure 2. The PSE, being defined as the arithmetic mean, differs from the St (being equal to the geometric mean) by a factor equal to $\sqrt{\mathrm{GV}\left(X_{St}\right)}$ (Figure 2A). Because this factor is always greater than 1, the use of the conventional measures suggests an absolute CE that is positive and grows linearly with St, the more so the wider the distribution (Figure 2B):

(4) $$\begin{array}{ll} \text{abs}.\;CE_{\text{A}} & =PSE_{\text{A}}-St=\;\left(\sqrt{\text{GV}(X_{St})}-1\right)\;\cdot \;St>0\;. \end{array}$$

The relative CE, defined as $\text{rel}.\;CE_{\text{A}}=\frac{\text{abs}.\;CE_{\text{A}}}{St}$, is therefore also positive. It is equal to the slopes of the lines in Figure 2B and independent of *St*. The use of *CE*_A_ as a measure of accuracy therefore implies the existence of positive deviations from *St* even though the subject was perfectly accurate; the positive values of *CE*_A_ are of purely mathematical origin. This is important to realize in, for example, studies of perceptual illusions. When positive values of *CE*_A_ are obtained, it is not justified to infer the existence of an illusion or bias, because the positive values may be a result of the convexity bias. The measure of accuracy that we propose is the geometric constant error *CE*_G_, which we derive in the section as the geometric counterpart of rel. *CE*_A_. In the special case assumed in the Gedanken experiment presented above, it yields a value of $CE_{\text{G}}=\ln\left(\frac{\mathrm{GM}\left(X_{St}\right)}{St}\right)=0$ (not shown in Figures 2A,B), which correctly indicates that the subject was perfectly accurate.

In the more general case, a subject may not be perfectly accurate. The center of the multiplicative normal distribution in our Gedanken experiment would then be $\mathrm{GM}\left(X_{St}\right)=St\cdot {\text{e}}^{\mu_{0}}$, where ${\text{e}}^{\mu_{0}}$ represents the positive error factor by which the center deviates from *St*. Therefore, $\ln\left(X_{St}\right)\sim N\left(\mu +\mu_{0},\sigma^{2}\right)$ with μ = ln(*St*). Our proposed measure of accuracy yields a value of $CE_{\text{G}}=\ln\left(\frac{\mathrm{GM}\left(X_{St}\right)}{St}\right)=\mu_{0}$. As shown in Figure 2C, rel. *CE*_A_ and *CE*_G_ can both be positive (quadrant I) or both be negative (quadrant III), but it is also possible that rel. *CE*_A_ is positive while *CE*_G_ is negative (quadrant II). The rel. *CE*_A_ does not fulfill the purpose of a measure of accuracy when data follow a multiplicative normal distribution, because it does not always reflect the true sign and never reflects the true magnitude of the deviation from *St*.

The second major goal of our study was, therefore, to derive novel definitions of the psychophysical measures using geometric descriptive statistics and the log-ratio distance function dist_ℝ>0_ that are consistent with positive-real physical quantities and that are not affected by the convexity bias. Other potential sources of bias, such as deviations of the distributions of matches from the multiplicative normal distribution, stimulus order, memory or attentional effects, or true perceptual illusions, may still remain and be reflected by the novel measure of accuracy. The proposed, geometric measures are summarized in Table 1 along with the conventional, arithmetic measures.

The third major goal of our study was to compare the outcomes of the analyses of our data using the conventional and the proposed novel definitions. We will show that the proposed definitions yield more homogeneous results and reveal effects of the carrier on accuracy and precision that are missed by the conventional definitions. We also find for our data that the conventional and proposed measures of accuracy have opposite signs.

## 2. Methods

### 2.1. Listeners

Twenty-two listeners participated in the study, with ten in Experiment 1 (six male and four female, ages 23–28 years), four in Experiment 2 (two male and two female, ages 25–29 years), and eight in Experiment 3 (four male and four female, ages 21–29 years). Listeners were informed about the purpose of the study, gave their written consent to participate, and were remunerated on an hourly basis. Twelve listeners had participated in other experiments in our lab during which detection and discrimination thresholds were measured using forced-choice procedures, but none had participated in experiments using the method of adjustment. For each listener, audiograms were measured prior to the experiments by means of standard audiometry (Otometrics Madsen Itera II) and found to be normal for both ears. The study was approved by the ethics committee of the Otto von Guericke University, Magdeburg.

### 2.2. Stimuli

Each acoustic stimulus consisted of an onset during which the amplitude of the stimulus rose linearly from zero to the maximum, a plateau during which the amplitude remained constant, and an offset during which the amplitude decayed linearly from the maximum to zero. Depending on the experiment, stimuli could differ in four attributes: onset duration, total duration, carrier, and sound level. They were generated in MATLAB R2010a (The Mathworks, Natick, MA, USA) and stored in the Waveform Audio File Format (WAVE) for replay by custom-made stimulus-presentation software. The system was calibrated using defined sound sources (Microtech Gefell MG 4000 and Brüel and Kjaer pistonphone 4228) and an artificial ear (Brüel and Kjaer 4153) equipped with a condenser microphone (Brüel and Kjaer 4133) connected to a conditioning amplifier (Brüel and Kjaer Nexus) and a multimeter (Agilent 34401A). During presentation, stimulus levels were controlled by a programmable attenuator (PA5, Tucker-Davis Technologies) linked downstream to a 24-bit sound card (Juli@, ESI Audiotechnik GmbH) of a desktop computer.

#### 2.2.1. Experiment 1

The onset durations (rise times) of the standard stimuli were between 0.5 and 64 ms, and neighboring values differed by a factor of 2. The onset durations of the comparison stimuli were between 0.25 and 128 ms long, and neighboring values differed by a factor of 2^1/6^ ≈ 1.1225. The offset duration of each stimulus was 50 ms. The total duration of each stimulus was 400 ms such that the plateau duration covaried with the onset duration. Two different carriers were used, a 3125-Hz tone and frozen white Gaussian noise. The stimuli were presented at sensation levels of 10, 22, and 34 dB SL.

#### 2.2.2. Experiment 2

Total stimulus durations were 200, 400, or 800 ms, including an offset duration of 50 ms. Onset durations of the standard stimuli were 2 ms and 64 ms. As in Experiment 1, the onset durations of the comparison stimuli were between 0.25 and 128 ms, and neighboring values differed by a factor of 2^1/6^ ≈ 1.1225. The carriers were a 3125-Hz tone and frozen white Gaussian noise. The stimuli were presented at sensation levels of 10 and 34 dB SL.

#### 2.2.3. Experiment 3

The total duration of all stimuli was 480 ms, including an offset duration of 48 ms. The onset duration of all standard stimuli was 10.24 ms. The possible onset durations of the comparison stimuli depended on the degree of range restriction. The smallest degree of restriction (degree 1) corresponded to the broadest range from 0.64 to 163.84 ms. The largest degree of restriction (degree 7) yielded the narrowest range from 6.64 to 15.79 ms. For all ranges (see Table 2), neighboring values differed by a factor of 2^1/16^ ≈ 1.0443. The carrier was a 3125-Hz tone. The stimuli were presented at a sensation level of 30 dB SL.

**Table 2:** **Ranges for the comparison stimuli used in Experiment 3**.

**Degree**	**Total range (ms)**	**Initial range < St (ms)**	**Initial range > St (ms)**
1	0.64–163.84	2.56–5.12	20.48–40.96
2	0.95–110.94	3.04–5.58	18.78–34.44
3	1.40–075.12	3.78–6.36	16.49–27.74
4	2.06–050.87	4.50–6.93	15.12–23.32
5	3.04–034.44	5.58–7.56	13.87–18.78
6	4.50–023.32	6.64–8.25	12.72–15.79
7	6.64–015.79	8.25–9.39	11.17–12.72

*In Experiment 3, the degree of range restriction determined the total range of adjustable onset durations and the initial ranges below and above the onset duration of the St (10.24 ms) from which the onset duration of the Co was randomly selected on the first trial*.

### 2.3. Procedures

#### 2.3.1. General procedures

Listeners sat on a comfortable chair within an illuminated and ventilated doubled-walled soundproof chamber (IAC Acoustics). Their task was to adjust a comparison stimulus to match a standard stimulus. In each trial of an experimental condition, a pair of stimuli consisting of the standard stimulus and the comparison stimulus, separated by a silent interval of 300 ms, was presented diotically using circumaural headphones (Sennheiser HDA 200). In parallel, a computer screen displayed a window with four buttons: one for increasing and one for decreasing the onset duration of the comparison stimulus, one for reporting a match, and one for starting, pausing, and continuing the experiment. Depending on individual preference, listeners could use a computer mouse or a keyboard to activate the buttons.

In each trial in each experiment, standard and comparison stimuli had the same carrier, the same total duration, and the same sound level, but differed in onset duration. In Experiments 1 and 2, the onset duration of the standard stimulus was selected at random from the list without replacement. The onset duration of the comparison stimulus in the first trial was selected at random from a list containing the products of the standard onset durations and the factors between 2^−12/6^ and 2^−6/6^ and between 2^+6/6^ and 2^+12/6^. In Experiment 3, the onset duration of the first comparison stimulus was selected at random from ranges below and above the standard, as specified in Table 2. In each experiment, the listener could use the up- and down-arrow buttons, either once or multiple times in quick succession, to increase or decrease the onset duration of the comparison stimulus to be presented in the next trial. Each button press altered the onset duration by a factor of 2^±1/6^ (in Experiments 1 and 2) or 2^±1/16^ (in Experiment 3). The standard was not changed. Listeners could repeat this adjustment process as often as they liked. When they perceived the standard and comparison stimuli to be the same, they indicated it with a button press. No feedback was provided. Next, a different onset duration for the standard stimulus (Experiments 1 and 2) or degree of range restriction (Experiment 3; see Table 2) was selected and the procedure was repeated. The random selection of the initial onset duration of the comparison stimulus from the ranges specified above made it unlikely that listeners adopted simple procedural strategies to solve the task.

To complete a given experiment, every listener had to come into the lab for multiple sessions over several months. A session lasted 2–2.5 h, including one or two breaks, as desired by the listener. Before the first training session started, the listeners' audiograms were measured. In Experiments 1 and 2, each listener underwent two guided training sessions with feedback to become familiar with the task and the stimuli. Detection thresholds for tone and noise stimuli were also measured, using a modified staircase procedure. The threshold measurements were repeated at the beginning of every fifth experimental session, because thresholds may vary from day to day (Hempstock et al., 1966; Heil et al., 2006). The thresholds were used to determine the attenuation required to achieve the desired sensation levels. Level adjustments were made when necessary. In Experiment 3, each listener underwent one guided training session with feedback prior to the first experimental session. Each subsequent session began with a training block to remind listeners of the task. The experimental blocks were preceded by detection-threshold measurements for tone stimuli, using a 3-alternative-forced-choice procedure. The thresholds were used to determine the attenuation required to achieve the desired sensation level of 30 dB SL.

#### 2.3.2. Structure of sessions

##### 2.3.2.1. Experiment 1

A session in Experiment 1 was constructed as follows. The sensation level of the initial standard stimulus was 34 dB SL, to start the session with the most clearly audible stimuli. The carrier (tone or noise) of the initial standard stimulus was selected at random to minimize order effects. The onset duration was also selected at random from the list of eight values (0.5, 1, 2, 4, 8, 16, 32, and 64 ms). The onset duration for the next standard stimulus was then drawn from the remaining seven values, and so on, until all eight standard onset durations had been drawn once, without changing the carrier or the sound level. Then, the next lower sound level (22 dB SL) was selected and the procedure repeated until all standard onset durations had been drawn once. Finally, the lowest sound level (10 dB SL) was selected and the procedure repeated until all standard onset durations had been drawn once. The entire procedure was then repeated for the other carrier, using all combinations of onset durations and sound levels. The entire procedure was repeated once more for each carrier, starting with the first. In this way, a listener made 96 adjustments in a single experimental session, with two adjustments for each of the 48 conditions (8 standard onset durations × 3 sound levels × 2 carriers). Each of the 10 listeners completed between 18 and 31 such sessions and therefore made between 36 and 62 adjustments for each of the 48 conditions (between 1728 and 2976 adjustments total).

##### 2.3.2.2. Experiment 2

A session in Experiment 2 was constructed as follows. The sensation level of the initial standard stimulus was 34 dB SL, to start the session with the most clearly audible stimuli. The carrier (tone or noise) of the initial standard stimulus was selected at random to minimize order effects. The onset duration (2 or 16 ms) and total duration (200, 400, or 800 ms) were also selected at random. The remaining onset duration was used for the next standard stimulus, while the total duration was unchanged. After both onset durations had been drawn once, the next total duration was selected, and so on, until all six combinations of onset and total durations had been presented four times. This whole procedure was then repeated for the sound level of 10 dB SL. The listener was encouraged to take a break of about 10 min, after which the whole procedure was repeated for the other carrier. In this way, a listener made 96 adjustments in a single experimental session, with four adjustments for each of the 24 conditions (2 standard onset durations × 3 total durations × 2 carriers × 2 sound levels). Each of the four listeners completed between 11 and 17 such sessions and therefore made between 44 and 68 adjustments for each condition (between 1056 and 1632 adjustments total).

##### 2.3.2.3. Experiment 3

A session in Experiment 3 began with a training block. Each experimental block was preceded by the detection-threshold measurements. An experimental block consisted of three or four repetitions of the seven randomly permuted degrees of range restriction, such that one experimental block consisted of 21 or 28 trials. The listeners performed as many experimental blocks as possible (usually four) within about 2 h of measurement time. Each of the eight listeners made between 74 and 110 adjustments for each of the seven degrees of range restriction (between 518 and 770 adjustments total).

### 2.4. Data analysis

All data analyses were performed using MATLAB R2010a (The Mathworks, Natick, MA, USA) with the Curve Fitting Toolbox, the Optimization Toolbox, the Parallel Computing Toolbox, and the Statistics Toolbox. In all statistical tests the α-criterion was 0.05.

## 3. Results

### 3.1. Onset-duration matches are consistent with a multiplicative model

**Figure 3:**
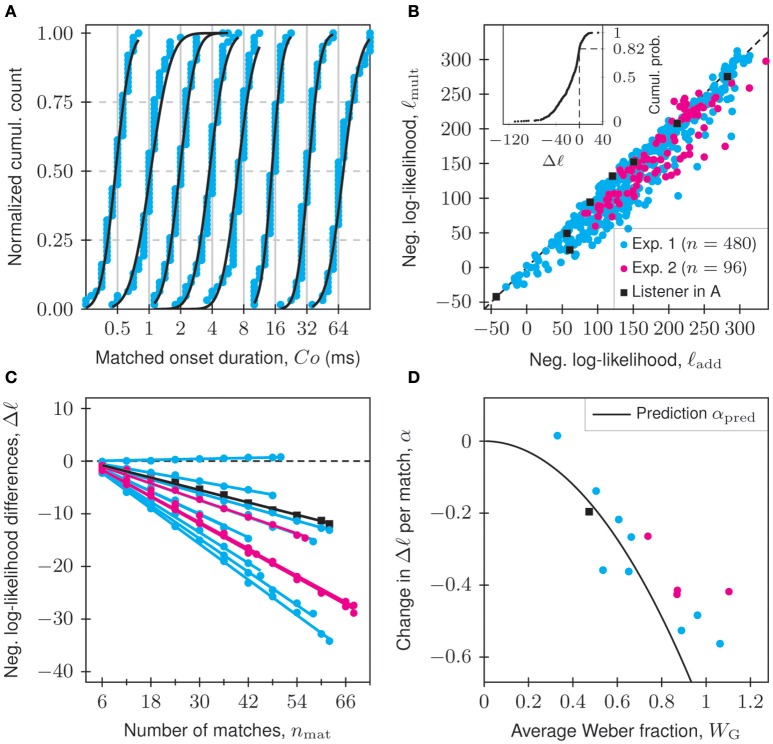
**Onset-duration matches are consistent with a multiplicative model. (A)** Matches (blue dots; *n*_mat_ = 62 each) for eight standard onset durations (gray vertical lines) of tones at 34 dB SL, plotted cumulatively for an example listener (L2) in Experiment 1, along with fitted multiplicative normal distributions (black lines). **(B)** Negative log-likelihoods of the fitted additive normal (ℓ_add_) and multiplicative normal (ℓ_mult_) distributions, plotted against each other for all 576 distributions of matches from Experiments 1 (*n* = 480, blue dots) and 2 (*n* = 96, pink dots). The data from the example listener and conditions in **(A)** are indicated by black squares. For data below the diagonal (black dashed line), the multiplicative normal distribution fits better. The inset, which plots the cumulative probability of the differences of negative log-likelihoods, Δℓ = ℓ_mult_ − ℓ_add_, shows that this is true for 82% of the data points. The median of Δℓ (−11.47) is significantly different from zero (Wilcoxon signed rank test; *p* = 5 · 10^−61^; two-tailed). **(C)** Dependence of Δℓ on the number of matches, *n*_mat_, included in the fitting process. Each dot represents the arithmetic average of a listener's Δℓ-values across all conditions of the corresponding experiment. Solid lines represent fits to Δℓ = α · (*n*_mat_ − 2), where the slope α is a free parameter. **(D)** Slope α of the best straight-line fit for each listener in **(C)** vs. the geometric average of the proposed Weber fractions *W*_G_, showing that the change in Δℓ per match is inversely related to precision. The black line is the relationship predicted under the assumption that matches follow perfect multiplicative normal distributions (see Supplementary Material for a derivation of α_pred_). The legend in **(B)** also applies to **(C,D)**.

According to our theoretical considerations, onset durations should be treated as elements of the multiplicative Euclidean vector space ℝ_>0_ rather than of the additive Euclidean vector space ℝ. To investigate empirically whether the matches of onset durations are consistent with an additive or with a multiplicative statistical model, we used the maximum-likelihood method to fit additive normal and multiplicative normal distributions to the distributions of matches obtained in Experiments 1 and 2, separately for each condition and listener (576 distributions in total, each based on between 36 and 68 matches). Even if other distributions might fit a particular dataset better than the additive normal and multiplicative normal distributions do, these two represent the simplest distributions describing data that behave according to an additive or a multiplicative Gaussian error model (Limpert et al., 2001; Limpert and Stahel, 2011). In fact, they are the maximum-entropy distributions when only the mean and the variance are known (Park and Bera, 2009). Fitting these two distributions therefore suffices to distinguish between additive and multiplicative models as long as no other constraints are imposed. The results of the fits are summarized in Figure 3.

Figure 3A shows, for one example listener in Experiment 1, the matches (blue dots; *n*_mat_ = 62 per *St*) for each of the eight standard onset durations (gray vertical lines) of tone stimuli presented at 34 dB SL. Superimposed are the cumulative distribution functions (CDFs) of the fitted multiplicative normal distributions (black lines). They fit the data well.

Figure 3B is a scatterplot of the negative log-likelihoods from the fits to the multiplicative normal distribution, ℓ_mult_, and to the additive normal distribution, ℓ_add_, for all conditions and listeners in Experiments 1 and 2. The data from the example listener and conditions in Figure 3A are indicated separately. The smaller ℓ, the better the fit. In 471 of 576 cases (82 %), the multiplicative normal distribution fits the data better than the additive normal distribution. This is illustrated more clearly in the inset of Figure 3B, which shows the cumulative distribution of the differences between the negative log-likelihoods, Δℓ = ℓ_mult_ − ℓ_add_. Negative values of Δℓ support the multiplicative normal distribution, whereas positive values support the additive normal distribution. Dashed lines indicate the fraction of negative differences (0.82). The median of Δℓ (−11.47) is significantly different from zero (Wilcoxon signed rank test; *p* = 5 · 10^−61^; two-tailed).

Because previous studies have obtained only few matches per listener (e.g., two matches in van Heuven and van den Broecke, 1979; van den Broecke and van Heuven, 1983), we also tested how the number of matches included in the fits influences the support for each model. From the distribution of matches for each condition and listener, we randomly drew a number of matches (*n*_mat_ ∈ {6, 12, 18, 24, …} or all), fitted them with additive normal and multiplicative normal distributions using the maximum likelihood method, and computed Δℓ. To reduce the noise, we arithmetically averaged the Δℓ across all conditions for a given *n*_mat_ and listener. Figure 3C plots these averages of Δℓ (dots) as a function of *n*_mat_, separately for each of the 14 listeners in Experiments 1 and 2. For all listeners but one, the multiplicative normal distribution is increasingly favored as additional matches are included. This shows that a sufficient number of matches per listener and condition is required for a clear empirical differentiation between the additive and the multiplicative models. Superimposed in Figure 3C (solid lines) are least-squares fits of the data from each listener to the straight-line equation Δℓ = α · (*n*_mat_ − 2) with *n*_mat_ ≥ 2. The offset was fixed to take into account that Δℓ = 0 for *n*_mat_ = 2. The slope α, which represents the change in Δℓ per match included in the fit, was a free parameter. For each listener, the slope is plotted in Figure 3D against the geometric average across all conditions of the geometric Weber fraction, *W*_G_ (see derivation in next section). The slopes are shallow for listeners with small average *W*_G_ (narrow distributions; high precision) and steep for listeners with large average *W*_G_ (broad distributions; low precision). The black line shows the relationship predicted under the assumption that all matches follow perfect multiplicative normal distributions. The data points fall near, though mostly above or to the right of, the prediction. Simulations (not shown) revealed that this is partly explained by averaging Δℓ and *W*_G_ from different distributions. In addition, the experimental data do not follow perfect multiplicative normal distributions.

In summary, when a sufficient number of matches is included, clear empirical support for the multiplicative model is obtained. This corroborates our theoretical considerations and the conclusion that onset-duration adjustment data should be described using geometric statistics and novel psychophysical measures, rather than arithmetic statistics and conventional psychophysical measures used in previous studies (e.g., van Heuven and van den Broecke, 1979; van den Broecke and van Heuven, 1983; Smurzyński and Houtsma, 1989). The novel measures are derived in the next section.

### 3.2. Derivation of psychophysical performance measures for positive-real quantities

Here, we derive probabilistic definitions of the psychophysical performance measures for the method of adjustment when the physical stimuli used can have only positive real magnitudes. We base our derivation on the (one-dimensional) Euclidean vector space ℝ_>0_ in which multiplication and exponentiation take on the role of vector addition and scalar multiplication and distances between any two elements *x, y* ∈ ℝ_>0_ are measured with the log-ratio distance function ${\mathrm{dist}}_{{\mathbb{R}}_{>0}}\left(x,y\right)=|\;\ln\left(x\right)-\ln\left(y\right)\;|=|\ln\left(\frac{x}{y}\right)|$. To obtain the operations and properties of ℝ_>0_, one can exploit the fact that ℝ and ℝ_>0_ are isometrically isomorphic, meaning that there are bijective functions from one space to the other that preserve the algebraic and metric structure. The most natural choice of bijections is the exponential function $\exp:\mathbb{R}\to {\mathbb{R}}_{>0},x\mapsto {\text{e}}^{x}$ and the logarithmic function ln:ℝ_>0_ → ℝ, *x* ↦ log_e_(*x*), where e ≈ 2.71828 is the Euler-Napier number. The Euclidean vector spaces ℝ and ℝ_>0_ are compared in Table 2 of the Supplementary Material.

For the method of adjustment, we are interested in calculating, for a given standard magnitude *St*, the constant and variable errors of a distribution of matches with positive-real magnitudes. To simplify matters, we assume that *X*_*St*_ approximately follows the canonical Gaussian distribution on ℝ_>0_, namely the multiplicative normal distribution, for which the relationships between parameters and statistical measures are well known (see Table 3 of the Supplementary Material). Let μ_0_ and σ^2^ be the parameters representing a potential constant error and the variable error, respectively, and let μ = ln(*St*). Note that when *X*_*St*_ follows a multiplicative normal distribution, the logarithm of *X*_*St*_ follows an additive normal distribution with the same parameters, i.e., $\ln\left(X_{St}\right)\sim N\left(\mu +\mu_{0},\sigma^{2}\right)$. Conceptually, the point of subjective equality should be the center of *X*_*St*_, such that

(5) $$PSE_{\text{G}}={\text{ e}}^{\mu +\mu_{0}}=St\;\cdot \;{\text{e}}^{\mu_{0}}=\text{ GM}(X_{St})\;.$$

We propose to define the constant error as the directed log-ratio distance between the PSE and the St:

(6) $$CE_{\text{G}}=\ln\left(\frac{PSE_{\text{G}}}{St}\right)=\ln(PSE_{\text{G}})-\ln(St)=\mu_{0}\;.$$

This directed distance differs from dist_ℝ>0_ (*PSE*_G_, *St*) in that the sign of the error is retained. It is a dimensionless measure with the logarithmic auxiliary unit neper (Np), which can be converted to the more familiar decibel (dB), where 1 Np = (20/ln(10)) dB ≈ 8.686 dB. By analogy with the Euclidean space ℝ, where the magnitude of the comparison stimulus that is just-noticeably different from the St is typically chosen to be *Co* = *St* ± AS(*X*_*St*_), let us define

(7) $$Co=St\;⋇\text{ GS}(X_{St})\;,$$

where the symbol ⋇ denotes “times or divided by”. Precision can then be quantified by a dimensionless difference limen defined as the geometric standard deviation of *X*_*St*_:

(8) $$DL_{\text{G}}=\{\begin{array}{ll} Co/St, & Co\geq St\\ St/Co, & Co<St \end{array}=\text{ GS}(X_{St})\;.$$

Unlike the conventional difference limen, *DL*_A_ = AS(*X*_*St*_), which must be divided by *St* to yield a relative measure of sensitivity, the proposed difference limen could be used directly as such a measure, because it is already such a ratio. For this reason it would be more correct to refer to it as a ratio limen, but by analogy to the conventional measure, we use the term geometric difference limen.

Note, however, that *DL*_G_ ∈ ℝ_>1_, whereas we want Weber fractions to be elements of ℝ_>0_, so that they are comparable to conventional Weber fractions. One might be tempted to define a novel Weber fraction as *W*_G_ = |*DL*_G_ − 1| = |GS(*X*_*St*_) − 1| = |GCV(*X*_*St*_)|, which is the geometric coefficient of variation proposed by Kirkwood (1979). However, this would be inconsistent with the operations allowed on ℝ_>0_. Instead, we propose to use the logarithm of *DL*_G_,

(9) $$W_{\text{G}}=\ln(DL_{\text{G}})={\text{ dist}}_{{\mathbb{R}}_{>0}}\;\left(Co,St\right)=\sigma \;,$$

because σ ∈ ℝ_>0_ alone satisfies the properties required of the Weber fraction as a measure of relative sensitivity. Again, the log-ratio distance between *Co* and *St* is a dimensionless quantity with the auxiliary unit neper, which can be converted to decibels. Due to their common auxiliary units, *CE*_G_ and *W*_G_ can be directly compared. The proposed geometric measures are contrasted with the conventional measures in Table 1, and we will use our experimental results to compare both types of measures in the sections that follow.

### 3.3. Dependence of accuracy and precision on onset duration, sound level, and carrier of the standard

The purpose of Experiment 1 was to examine how accuracy and precision of onset-duration matching depend on the onset duration, the sound level, and the carrier of the standard and to explore inter-listener variability. We compare the results when accuracy and precision are quantified by the conventional and the proposed measures. We find quantitative and qualitative differences which may even lead to opposite conclusions.

#### 3.3.1. Inter-listener variability

**Figure 4:**
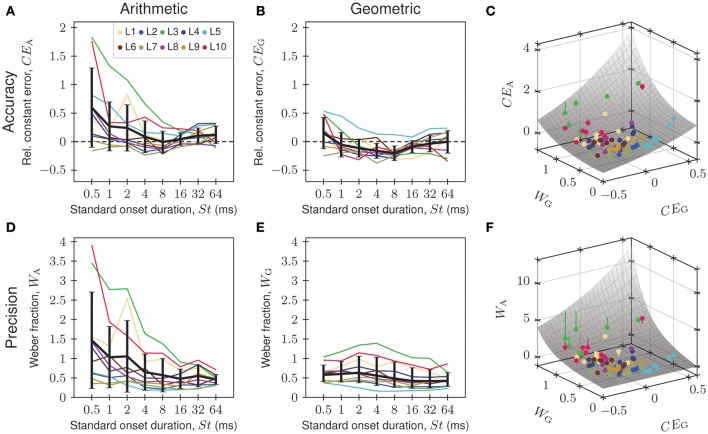
**(A,B,D,E)** Dependence of accuracy (top row) and precision (bottom row) of onset-duration matching on the onset duration of the standard, when using the conventional (left column) and our proposed (middle column) psychophysical measures. Data were obtained with tone stimuli presented at 22 dB SL. Solid colored lines represent different listeners (see legend in **A**), and solid black lines and error bars represent the averages and standard deviations across all listeners (AM ± AS in **A,B,D**; GM ⋇ GS in **E**). **(C,F)** Arithmetic constant errors **(C)** and arithmetic Weber fractions **(F)** as functions of geometric constant errors and geometric Weber fractions in three-dimensional plots. Same color code as in the other panels. The gray surfaces represent the relationships predicted for perfect multiplicative normal distributions (see Supplementary Material for a derivation). The colored vertical lines show how far the data points are off the surface in the vertical direction. The three functions in Figure 2C correspond to slices through the surface in **(C)** at *W*_G_ values of $\sqrt{0.2}$, $\sqrt{0.4}$, and $\sqrt{0.6}$.

Figure 4 shows, as an example, the constant error and the Weber fraction as a function of the onset duration of the standard for tones presented at 22 dB SL. Each colored line represents the data from a given listener (*n* = 10; see legend of Figure 4A), whereas the black solid lines and error bars represent the averages and standard deviations across listeners.

As shown in Figure 4A, the conventional measure of accuracy, the relative constant error *CE*_A_, is usually positive, suggesting that listeners overestimate onset duration. The magnitude of *CE*_A_ tends to decrease with increasing onset duration of the standard. Furthermore, at short onset durations, values of *CE*_A_ differ widely among listeners (varying from −0.16 to 1.83) whereas at long onset durations, values are more similar (varying only from −0.11 to 0.32). This heteroscedasticity is reflected in the systematic and pronounced decrease of the arithmetic standard deviation of *CE*_A_, as well as of the arithmetic mean of *CE*_A_, with increasing onset duration of the standard. The null hypothesis of equal variance cannot be maintained (Bartlett test; *p* < 10^−6^; *df* = 7).

In contrast, and as shown in Figure 4B, the proposed measure of accuracy, the relative constant error *CE*_G_, is most often negative, revealing that listeners tend to underestimate onset duration. Only for listener L5 is *CE*_G_ consistently positive. The measure tends to be positive also for the shortest onset duration of the standard, an effect that may be attributed to the boundary effects imposed on the distribution of matches by the restricted range of comparison stimuli (see results of Experiment 3 below). Furthermore, the variation of *CE*_G_ across listeners is similar at all onset durations of the standard, and extreme values, as seen for *CE*_A_ at short onset durations, are not observed. This homoscedasticity of *CE*_G_ is reflected in similar arithmetic standard deviations of *CE*_G_ at all onset durations of the standard. The null hypothesis of equal variance of *CE*_G_ can be maintained (Bartlett test; *p* = 0.0947; *df* = 7). The variation of the arithmetic mean of *CE*_G_ with the onset duration of the standard is also smaller than the variation of the arithmetic mean of *CE*_A_ (cf. Figures 4A,B).

Similar observations were made for precision (Figures 4D,E). At short onset durations, values of the conventional measure of precision, the Weber fraction *W*_A_, differ widely among listeners (from 0.35 to 3.91) whereas at long onset durations, values are more similar (varying only from 0.30 to 0.71). Again, this high degree of heteroscedasticity is reflected in the systematic and pronounced decrease of the arithmetic standard deviation of *W*_A_ with increasing onset duration of the standard, and the null hypothesis of equal variance of *W*_A_ cannot be maintained (Bartlett test; *p* < 10^−6^; *df* = 7). The heteroscedasticity is also reflected in the monotonic decrease of the arithmetic mean of *W*_A_, from about 1.5 for 0.5 ms to about 0.5 for 64 ms (Figure 4D). Also note that the arithmetic mean and arithmetic standard deviation do not characterize these data well. For example, only three listeners have larger, but seven listeners have lower, *W*_A_ values than the mean. Also, at several onset durations of the standard, the lower bound of the arithmetic standard deviation falls below the lowest *W*_A_ observed. These oddities arise because data that cannot be additive-normally distributed (*W*_A_ is always positive) have been characterized by arithmetic statistical measures.

In contrast, and as shown in Figure 4E, variation across listeners of the proposed measure of precision, the Weber fraction *W*_G_, is similar at all onset durations of the standard. This homoscedasticity of *W*_G_ is reflected in similar geometric standard deviations of *W*_G_ at all onset durations of the standard. The null hypothesis of equal variance of *W*_G_ can be maintained (Bartlett test; *p* = 0.5816; *df* = 7). Furthermore, in individual listeners, the deviations of *W*_G_ from Weber's law are much smaller than for *W*_A_, and extreme values, as seen for *W*_A_ at short onset durations, are not observed. Also, the geometric mean of *W*_G_ varies much less with the onset duration of the standard (from about 0.4 to 0.6) than the arithmetic mean of *W*_A_ (cf. Figures 4D,E). Note that the geometric mean and the geometric standard deviation characterize these data well.

We also observed that the order of the listeners with respect to accuracy or precision can differ, depending on whether arithmetic or geometric measures are used. For instance, when *CE*_A_ is used, listener L3 appears to be the least accurate (Figure 4A), whereas when *CE*_G_ is used, listener L5 is the least accurate (Figure 4B). This can happen because the relationship between *CE*_A_ and *CE*_G_ is not order-preserving. Instead, *CE*_A_ depends on both *CE*_G_ and *W*_G_. The gray surface in Figure 4C represents the relationship predicted for perfect multiplicative normal distributions (see Supplementary Material for a derivation). A large *CE*_A_ can result from a large *W*_G_ even when *CE*_G_ is small (e.g., as is the case for listener L3; see Figure 4E). Similarly, the relationship between *W*_A_ and *W*_G_ is also not order-preserving, because *W*_A_ also depends on both *CE*_G_ and *W*_G_. The gray surface in Figure 4F represents the relationship predicted for perfect multiplicative normal distributions (see Supplementary Material for a derivation). The colored points in Figures 4C,F are derived from the data shown in Figures 4A,B,D,E (same color code). The colored vertical lines represent the vertical distances of the observed *CE*_A_ and *W*_A_ from the surfaces. Most of them are short (not exceeding the symbols representing the data), such that the observed *CE*_A_ and *W*_A_ are generally close to those that would be obtained from perfect multiplicative normal distributions. These analyses corroborate our finding that the distributions of matches are well described by multiplicative normal distributions (cf. Figure 3).

#### 3.3.2. Grand-mean data

**Figure 5:**
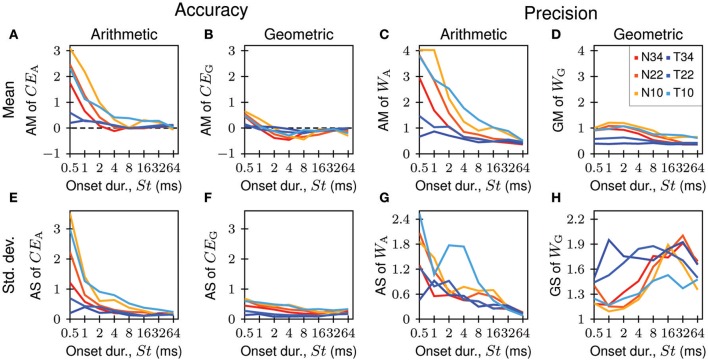
**Comparison of the conventional (arithmetic) and our proposed (geometric) constant errors and Weber fractions as measures of accuracy and precision, respectively**. Shown are the means **(A–D)** and standard deviations **(E–H)** computed across all 10 listeners. Each line represents data for a different combination of carrier and sound level (see legend in **D**; the letters identify the carrier and the numbers the sensation level).

The results described so far, based on tones at 22 dB SL, apply in a qualitatively similar fashion to tones at the other sound levels and to the noise carrier. This is illustrated in Figure 5. Figure 5A shows the conventional measure of accuracy, the relative constant error *CE*_A_, as a function of the onset duration of the standard. Each line represents the arithmetic mean of the *CE*_A_ across all 10 listeners for the tone or noise carrier at a given sound level (see legend in Figure 5D). The function for tones at 22 dB SL is identical to the mean function in Figure 4A. For both carriers and at all sound levels, the arithmetic mean of *CE*_A_ is predominantly positive and decreases with increasing onset duration of the standard in a manner that varies with sound level and carrier (see below). Figure 5E shows the corresponding arithmetic standard deviations of *CE*_A_. For both carriers and all sound levels, the arithmetic standard deviation of *CE*_A_ decreases with increasing onset duration of the standard, reflecting the fact that the variance of *CE*_A_ across listeners is heterogeneous. The heteroscedasticity reflected in these curves decreases with increasing sound level for a given carrier. For a given sound level, the heteroscedasticity is more pronounced for the noise than for the tone carrier, but the null hypothesis of equal variance of *CE*_A_ cannot be maintained for any of the six combinations of sound level and carrier (Bartlett tests; for all *p* < 0.0025 and *df* = 7).

In contrast, and as shown in Figure 5B, the arithmetic mean of *CE*_G_ is mostly negative, except at the two shortest onset durations of the standard. Again, this may be attributed to the boundary effects imposed on the distribution of matches by the restricted range of comparison stimuli (see results of Experiment 3 below). Furthermore, extreme values of the constant error such as those seen with *CE*_A_ are not observed for *CE*_G_. Also, the variation of *CE*_G_ with the onset duration of the standard is much less pronounced than that of *CE*_A_ (cf. Figures 5A,B). The arithmetic standard deviation of *CE*_G_ changes little with the onset duration of the standard, reflecting a high degree of homoscedasticity (Figure 5F). Only for the noise carrier at 10 dB SL and 22 dB SL can the null hypothesis of equal variance of *CE*_G_ not be maintained (Bartlett test; *p* = 0.0128 and *p* = 0.0064, respectively; *df* = 7). For a given carrier, the arithmetic standard deviation of *CE*_G_ decreases with increasing sound level. For a given sound level, it is lower for the tone than for the noise carrier.

Figures 5C,D,G,H show the corresponding results for precision. Figure 5C shows the pronounced decrease of the arithmetic means of the conventional measure of precision, the Weber fraction *W*_A_, with increasing onset duration of the standard for all sound levels and carriers. The function for 22 dB SL is the same as that shown in Figure 4D. This decrease is consistent with findings in the literature (cf. Figure 1) and implies that Weber's law does not hold under these conditions. The corresponding arithmetic standard deviations of *W*_A_ also decrease with increasing onset duration of the standard (Figure 5G), reflecting the high degree of heteroscedasticity of *W*_A_. The null hypothesis of equal variance of *W*_A_ cannot be maintained for any of the six combinations of sound level and carrier (Bartlett tests; for all *p* < 10^−4^ and *df* = 7).

In contrast, for both carriers and at all sound levels, the geometric mean of the proposed measure of precision, the Weber fraction *W*_G_, changes little with onset duration of the standard (Figure 5D), although only for tones at 34 dB SL can Weber's law be said to apply (Friedman test; *p* = 0.7643). For tones at the lower two sound levels and for the noise carrier at all sound levels, the geometric mean of *W*_G_ initially increases with increasing onset duration of the standard from 0.5 to 1 or 2 ms before decreasing with further increases of the onset duration of the standard. The initial increase may again be attributed to the boundary effects imposed on the distribution of matches by the restricted range of comparison stimuli (see results of Experiment 3 below). Without such boundary effects, *W*_G_ may be expected to only decrease with increasing onset duration of the standard, but only slightly compared to *W*_A_. The geometric standard deviation of *W*_G_ does not show the systematic decrease displayed by the arithmetic standard deviation of *W*_A_ (cf. Figures 5G,H). For tones at all sound levels and for the noise carrier at 34 dB SL, the null hypothesis of equal variance of *W*_G_ can be maintained (Bartlett tests; all *p* > 0.2265 and *df* = 7). For the noise carrier at 10 dB SL and 22 dB SL, the null hypothesis of equal variance cannot be maintained (Bartlett test; *p* = 0.0040 and *p* = 0.0361, respectively; *df* = 7).

#### 3.3.3. Effects of sound level and carrier

**Figure 6:**
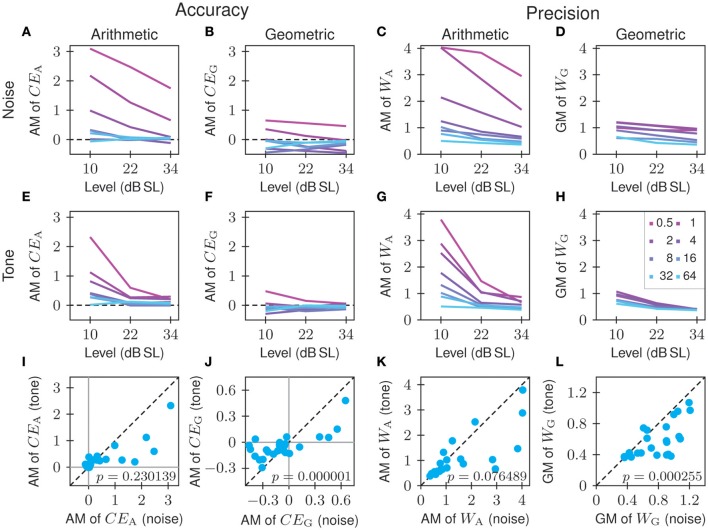
**Effects of sound level and carrier on onset-duration matching**. Top and middle rows: Constant errors and Weber fractions plotted against sound level separately for noise **(A–D)** and tone **(E–H)** stimuli. Each colored line corresponds to one onset duration of the standard (see legend in **H**; numbers in ms). Bottom row: Constant errors and Weber fractions for tone stimuli plotted against those for noise stimuli of corresponding onset duration and level of the standard **(I–L)**. Note that the conventional measures, but not the proposed measures, fail to reveal a significant effect of the carrier on accuracy and precision (*p*-values are for two-sided Wilcoxon paired signed rank tests).

The effects of sound level on accuracy and precision are summarized in the top and middle rows of Figure 6, in which the mean data from Figure 5 are replotted. The top row shows the data for the noise carrier and the middle row shows those for the tone carrier, plotted as functions of the sound level with each colored line corresponding to one of the eight onset durations of the standard (see legend in Figure 6H). Generally, accuracy and precision increase with increasing sound level, as reflected in the decrease of the magnitudes of the constant error (cf. Figures 6A,B,E,F) and the Weber fraction (cf. Figures 6C,D,G,H). The poorer the accuracy and precision for the lowest sound level, the stronger is the improvement with sound level. The effects of sound level appear more pronounced and more dependent on the onset duration of the standard for the conventional (arithmetic) than for the proposed (geometric) measures.

The effect of the carrier on accuracy and precision are summarized in the bottom row of Figure 6, in which the mean of the measures for the tone carrier are plotted against those for the noise carrier of corresponding sound level and onset duration of the standard. Notably, when the conventional measures are used, the type of carrier has no significant effect on accuracy (*p* = 0.2301; Wilcoxon paired signed rank test; two-sided) or precision (*p* = 0.0765). In contrast, when the proposed measures are used, significant effects of the carrier on both accuracy (*p* = 10^−6^) and precision (*p* = 2.5 · 10^−4^) are revealed.

### 3.4. Accuracy and precision of onset-duration matching do not systematically depend on plateau duration

The purpose of Experiment 2 was to explore whether listeners use differences in the plateau duration, rather than in the onset duration, of the standard and comparison stimuli for matching. In Experiment 1, the total duration (400 ms) and the offset duration (50 ms) were fixed, such that plateau duration covaried with onset duration. If listeners actually matched plateau duration, and if plateau-duration matching approximately followed Weber's law, then the performance of the apparent onset-duration matching would improve by shortening, and worsen by prolonging, the plateau duration of the standard. To test this idea, we employed standards with total durations of 400 ms (as in Experiment 1), 200 ms, and 800 ms. The offset duration was fixed at 50 ms (as in Experiment 1). Again, tone and noise carriers were used but with only two sound levels (10 dB SL and 34 dB SL) and only two onset-durations of the standard (2 and 16 ms).

**Figure 7:**
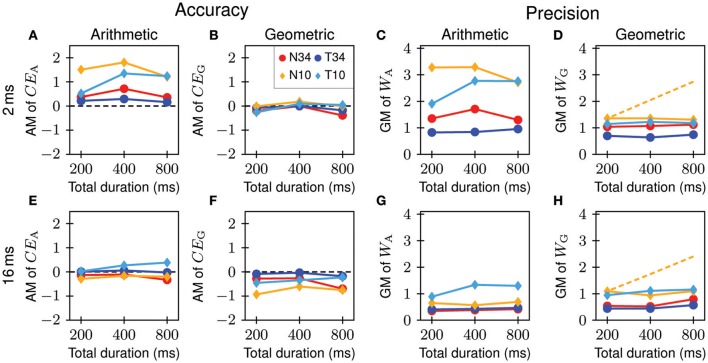
**Effects of plateau duration on arithmetic and geometric constant errors and arithmetic and geometric Weber fractions**. Each colored line connects data obtained for a given combination of onset duration, sound level, and carrier of the standard (legend in **B**; letters identify the carrier and numbers the sensation level). Top rows **(A–D)** and bottom rows **(E–H)** show the data for onset durations of the standard of 2 ms and 16 ms, respectively. Yellow dashed lines in **(D,H)** show the geometric Weber fractions, *W*_G_, predicted on the assumptions that listeners match the plateau duration rather than the onset duration and that *W*_G_ for plateau-duration matching is constant, using the *W*_G_ obtained for the noise carrier at 10 dB SL and the total duration of 200 ms as a reference.

Neither accuracy nor precision have a monotonic dependence on plateau duration (Figure 7). For the majority of combinations of sound level, carrier, and onset duration (7/8 for *CE*_A_; 5/8 for *CE*_G_; 8/8 for *W*_A_ and 6/8 for *W*_G_), the null hypothesis of equal medians can be maintained (Friedman test; *p* > 0.105). For the remaining combinations, *p*-values were 0.0498 (4/6) or 0.0388 (2/6). In most cases, however, accuracy and precision changed nonmonotonically. In addition, precision changed much less than predicted if listeners matched plateau durations in accordance with Weber's law (represented by the yellow dashed lines in Figures 7D,H). It is therefore unlikely that listeners performed Experiments 1 and 2 by matching plateau durations. It is likely that listeners instead use onset cues, specifically differences in onset duration *per se* or associated spectral differences (see Discussion).

The results of Experiment 2, obtained with four listeners who did not take part in Experiment 1, also corroborate the differences in the arithmetic and geometric measures of accuracy and precision described in the previous section (Figure 7). They also corroborate the major findings of Experiment 1: accuracy and precision are higher for the tone than for the noise carrier, accuracy and precision increase with increasing sound level for both carriers, estimates of the constant error are more positive for short (2 ms) than for long (16 ms) onset durations of the standard, and precision increases with increasing onset duration of the standard (Figure 7).

### 3.5. Effects of restricting the range of comparison stimuli

In Experiment 3, we examined the effects of restricting the range of adjustable comparison stimuli on the measures of accuracy and precision. The experiment was motivated by our observation in Experiments 1 and 2 that some of the distributions of matches for standards with short onset durations, low sound levels, and noise carriers, might have been subject to boundary effects due to restriction of the range of adjustable comparison stimuli. In Experiment 3, a single standard stimulus with an onset duration of 10.24 ms was used, and the range of adjustable comparison stimuli was increasingly restricted from values between 0.64 and 163.84 ms (first degree of restriction) to values between 6.64 and 15.79 ms (seventh degree of restriction; see Table 2).

**Figure 8:**
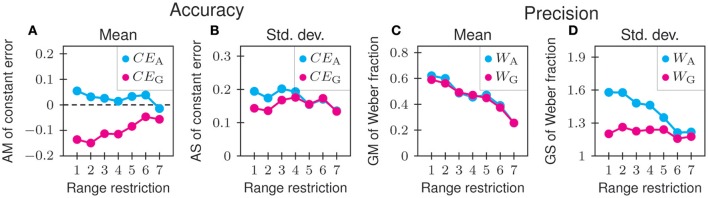
**Effects of restricting the range of adjustable comparison stimuli on the measures of accuracy and precision**. Shown are the arithmetic mean **(A)** and arithmetic standard deviation **(B)** of the conventional (*CE*_A_) and proposed (*CE*_G_) constant errors, as well as the geometric mean **(C)** and geometric standard deviation **(D)** of the conventional (*W*_A_) and proposed (*W*_G_) Weber fractions, as a function of the degree of range restriction.

Figure 8 shows the results of Experiment 3. The arithmetic mean of *CE*_A_ decreases with increasing range restriction from about 0.5 to about 0. The arithmetic mean of *CE*_G_ increases from about −0.15 and approaches 0 with increasing range restriction (Figure 8A). The absolute value of both measures of the constant error therefore diminishes as the range of adjustable comparison stimuli is restricted. The associated arithmetic standard deviation of *CE*_A_ also decreases with increasing range restriction, whereas that of *CE*_G_ shows no systematic change (Figure 8B). The geometric means of both *W*_A_ and *W*_G_ decrease monotonically with increasing range restriction, both from about 0.6 to 0.25 (Figure 8C). The decrease is expected because *W*_A_ and *W*_G_ are equal to 0 when only a single value of the comparison stimulus can be chosen. Precision therefore appears to improve with increasing range restriction. The geometric standard deviation of *W*_A_ decreases with increasing range restriction whereas that of *W*_G_ changes little over the seven degrees of range restriction we tested. Ultimately, however, the geometric standard deviations of *W*_A_ and *W*_G_ must approach 1 as the range of adjustable comparison stimuli is increasingly restricted. They are equal to 1 when only a single value of the comparison stimulus can be chosen. Boundary effects imposed on the distributions of matches by the restricted range of comparison stimuli might therefore underlie the positive values of *CE*_G_ for short onset durations of the standard, as illustrated in Figure 5B, and the initial increase in *W*_G_ with increasing onset duration of the standard for some combinations of tone level and carrier, as illustrated in Figure 5D.

## 4. Discussion

### 4.1. Convexity bias, illusions, and novel measures

We derived novel definitions of the psychophysical measures of the point of subjective equality, the constant error, the difference limen, and the Weber fraction for stimuli with positive-real magnitudes. Our aim was consistency: When two positive-real quantities are combined, then the result should also be positive real. This requirement is not always met when positive-real quantities are treated as if they were elements of the additive vector space of real numbers. The multiplicative Euclidean vector space of positive-real numbers endowed with the log-ratio distance function, however, fully satisfies this requirement. In a recent note, Graff (2014) promotes the use of the log-ratio distance function, arguing that relative differences between physical quantities are often more informative than absolute differences. Moreover, he makes the point that the determination of psychophysical laws first requires a clear-cut, objective expression of the physics of stimuli and responses. He shows that the log-ratio distance function exhibits all properties desired of a measure of relative differences, such as symmetry in selecting a reference, agreement between inverted units, and additivity. We build upon these ideas by linking the algebraic properties of the multiplicative Euclidean vector space with descriptive statistics. The geometric mean, unlike the arithmetic mean, is consistent with the structure of the multiplicative Euclidean space. Our Gedanken experiment (Figure 2) demonstrates that the conventional psychophysical measures possess a convexity bias of purely mathematical origin. As summarized in Figure 2C, the conventional arithmetic constant error would imply the existence of a positive bias or perceptual illusion (i.e., when *CE*_A_ > 0), whose magnitude increases with the width of the distribution of matches, when no bias or illusion exists (i.e., when *CE*_G_ = 0). A true bias or illusion (i.e., when *CE*_G_ ≠ 0) would be overestimated, missed, or underestimated, depending on its sign and magnitude and on the width of the distribution of matches. It is even possible, as in the present study, that *CE*_A_ > 0 while *CE*_G_ < 0 (see quadrant II in Figure 2C).

It may be criticized that the Gedanken experiment rests on the assumption that matches follow a multiplicative normal distribution and that the outcome may differ if the matches followed other distributions. However, the multiplicative normal distribution is the simplest model describing data that behave according to a multiplicative model (Limpert et al., 2001; Limpert and Stahel, 2011), and it is the maximum-entropy distribution on ℝ_>0_ when only the mean and the variance are known (Park and Bera, 2009). Furthermore, the multiplicative normal distribution may be difficult to distinguish from other multiplicative distributions, such as the Pareto distribution, a power-law distribution (Newman, 2005; Clauset et al., 2009). We chose the multiplicative normal distribution because it allowed us to exploit the known relationships between its parameters and the statistical descriptive measures (Table 3 of the Supplementary Material) and to analytically determine the magnitude of the convexity bias. The particular distribution on ℝ_>0_ is, however, not so relevant for the main argument: the convexity bias is a consequence of the AM-GM inequality, a special case of Jensen's inequality, and its existence is independent of the underlying distribution. The Gedanken experiment applies not only to the matching of onset durations, but also challenges matching studies in which the existence of perceptual illusions was inferred from, or their magnitudes were quantified by, the conventional constant error. This includes reports of the auditory time-stretching illusion (e.g., Sasaki et al., 2010) and also certain optical illusions such as the famous Müller-Lyer illusion (e.g., Restle and Decker, 1977; Bulatov et al., 1997; Bulatov and Bertulis, 1999; Woloszyn, 2010), the vertical–horizontal illusion (e.g., Higashiyama, 1996), and Titchener and Delboeuf illusions (e.g., Pressey, 1977). The impact of the convexity bias on measures of such perceptual illusions remains to be investigated. Another issue is how the theoretical considerations can be applied to other psychophysical methods, such as forced-choice procedures, where performance is quantified differently.

It may be argued that the problems we have identified above can be avoided if the conventional arithmetic analysis is performed on log-transformed data, as is often the case in the literature. Indeed, the geometric mean and standard deviation on which our proposed measures of accuracy and precision are based can be obtained by exp-transforming the arithmetic mean and standard deviation of the log-transformed random variable (see Table 1 of the Supplementary Material). However, log-transformation is often used only to make skewed distributions more symmetric (e.g., bell shaped), a requirement of many statistical tests. In hearing sciences, log-transformation of the stimulus amplitude (into decibels) serves mainly to compress the wide range of sound pressures (> six orders of magnitude) or sound intensities (> twelve orders of magnitude) that are processed by auditory systems into a more manageable range of values and to respect the observation that equal increments in level correspond to roughly equal increments in sensation (Hassall and Zaveri 1988, p. 31; Moore 2013, p. 10; Pickles 2013, p. 3). When to log-transform data, when to back-transform measures obtained in log-space, or how to proceed with the back-transformed measures may often be only vaguely known. Because, historically, concepts in descriptive statistics have usually been developed based on the additive Euclidean vector space of real numbers, their mathematical relationships to the multiplicative Euclidean vector space of positive-real numbers may be little known. To avoid biases that are of purely mathematical origin, it is essential to respect that corresponding mathematical operations differ between the vector space of real numbers and that of positive-real numbers. For example, addition corresponds to multiplication, multiplication corresponds to exponentiation, and the Euclidean distance function dist_ℝ_ (*x, y*) = |*x* − *y*| corresponds to the Euclidean distance function dist_ℝ>0_ (*x, y*) = |ln(*x*) − ln(*y*)|. Our study may be the first in the field of perception science to demonstrate the mathematical necessity for geometric measures of performance when stimulus magnitudes can only be positive real. Our approach extends beyond simply log-transforming data and using arithmetic statistics.

### 4.2. Accuracy and precision in onset-duration matching

We reexamined onset-duration matching with respect to stimulus factors affecting the accuracy and precision of adjustments, as well as with respect to inter-listener variability measured with the conventional and the proposed psychophysical measures. We demonstrated that the multiplicative normal distribution usually fits the distributions of matches better than the additive normal distribution (Figure 3). This is in line with our theoretical considerations according to which matches of onset durations, or of other stimulus attributes that can attain only positive real magnitudes, should comply with a multiplicative rather than an additive model. We also showed that the superiority of the fit of the multiplicative normal distribution over that of the additive normal distribution depends on the width of the empirical distribution and on the number of matches. Unlike previous studies which are based on only few matches per listener and condition (e.g., van Heuven and van den Broecke, 1979; van den Broecke and van Heuven, 1983), the listeners in our study made many (up to 68) matches per condition. This is clearly sufficient to demonstrate the superiority of the multiplicative model and the use of geometric rather than additive descriptive statistical measures to characterize the distributions of matches and to quantify performance.

It may be argued that the superiority of the multiplicative model, demonstrated here (Figure 3), results from the logarithmic spacing of the adjustable onset durations of the comparison stimuli and that opposite results would have been obtained with linear spacing. For the fitting procedure, however, it makes no difference how the samples are spaced. Nonetheless, it is conceivable that the spacing of the stimuli might affect the way listeners perform the tasks. For example, incrementing the onset duration by some amount above the standard constitutes a smaller relative change than decrementing it by the same amount below the standard. Such an asymmetry might bias the distribution of matches.

When comparing the behavior of the conventional and the proposed measures of accuracy and precision across listeners and conditions, we observed that the conventional measures display a high degree of heteroscedasticity and a strong dependence on the onset duration and the sound level of the standard. This would mean that the generalization gradients of performance differ markedly between listeners. In contrast, the proposed measures display a high degree of homoscedasticity and a weak dependence on the onset duration and the sound level of the standard (Figures 4–6), meaning that the generalization gradients of performance are similar for different listeners.

Furthermore, we found that the carrier of the standard has a significant influence on accuracy and precision of onset-duration matching, an effect detected when using the proposed geometric measures but missed with the conventional measures (Figure 6). We found that both accuracy and precision were better for the narrowband (tone) than the broadband (noise) carrier. This is consistent with the trend observed in the data of van Heuven and van den Broecke (1979) and van den Broecke and van Heuven (1983) and observed when comparing the findings of Kewley-Port and Pisoni (1984) with those of Smurzyński and Houtsma (1989) (see Introduction and Figure 1B). The better performance with narrowband than broadband carriers can be explained by assuming that listeners make use of additional spectral cues available when using the former. The spectral splatter induced by the onset of a tone increases with decreasing onset duration. These spectral differences might be exploited by the listeners, in addition to the temporal differences, when matching the onset durations of tones. With broadband carriers, the spectral differences are less pronounced or absent.

We found no systematic effect of total duration (and hence plateau duration) on accuracy or precision (Figure 7), suggesting that differences in plateau duration can be excluded as a dominant cue for onset-duration matching in our experiments. This is in line with a conclusion drawn by van Heuven and van den Broecke (1979). This leaves the temporal differences at the onset (and, as discussed above, their associated spectral differences) as potential cues. One crucial parameter may be the rate of rise of the amplitude at stimulus onset. This rate doubles when onset duration is halved and sound level is held constant. Under these conditions, accuracy and precision slightly decrease with increasing rate of rise, even with the geometric measures. On the other hand, the rate of rise of the amplitude at stimulus onset also increases when sound level is increased and onset duration is held constant. The rate of rise increases by a factor of four for a 12-dB increment in sound level. Under these conditions, however, accuracy and precision improve with increasing rate of rise. The rate of rise therefore cannot be the only factor. The onset responses of cortical and subcortical neurons are very sensitive to the rate of rise of the stimulus amplitude, as observed in many studies that have varied onset duration or sound level (Hall and Feng, 1988; Phillips, 1988; Gooler and Feng, 1992; Heil, 1997a,b; Heil and Irvine, 1998; Lee et al., 2016), but onset responses are not a unique function of this rate. Based on the effects of varying onset duration, onset shape, and sound level on the onset responses of cat auditory cortical neurons, Heil and colleagues suggested that the first spikes of the responsive population of neurons track the onset envelope and the initial steady-state portion of the stimulus (for review, see e.g., Heil, 2003). They also concluded that each temporal envelope will evoke a unique spatiotemporal response pattern across the tonotopic and isofrequency axes of auditory cortical maps. Such unique patterns may be the basis for (or a physiological correlate of) the onset-matching capabilities and their dependence on onset duration, level, and carrier of the standard reported here.

Notably, the sign of the constant error in our data was affected by the type of measure used. When using the conventional measure, the relative *CE*_A_, values were typically positive (Figures 4–8), implying that listeners overestimated onset durations. Our analyses, however, show that in our data positive values of *CE*_A_ are likely the result of the convexity bias. The proposed measure of accuracy, the geometric constant error *CE*_G_, was negative for most listeners and conditions. Values of *CE*_G_ tended to be positive only for the shortest onset duration of the standard, which might be due to a boundary effect imposed by the restricted range of comparison stimuli. The presence of predominantly negative values of *CE*_G_ may be due to presentation-order effects (Gescheider, 1997), given that the standard was always presented before the comparison stimulus. Whether this is a viable explanation could be assessed, if feasible, in future studies that also employ reversed and balanced (e.g., randomized) sequences of standard and comparison stimuli.

The results of Experiment 3 show that constant errors and Weber fractions converge toward zero when the range of values from which the comparison stimuli can be drawn is increasingly restricted (Figure 8). Thus, when the range of adjustable comparison stimuli is too narrow, the presence of systematic biases or illusions—no matter if positive or negative—will be missed, and precision will be overestimated. This supports a conclusion drawn by van Heuven and van den Broecke (1979), who argued that ceiling effects reduced the standard deviations computed from the distribution of matches to standards with the longest onset durations. A similar reduction of the standard deviations will occur for matches to standards with the shortest onset durations. Range effects therefore cannot be the origin of the rapid increase of conventional Weber fractions with decreasing onset duration at short onset durations of the standard.

### 4.3. Conclusions

Arithmetic descriptive statistics are appropriate for random variables over the additive Euclidean space ℝ, whereas geometric descriptive statistics should be used for random variables over the multiplicative Euclidean space ℝ_>0_, in which relative differences are measured with the log-ratio distance function. Our results imply that psychophysical studies should be reevaluated if arithmetic descriptive statistics were used to quantify performance when geometric statistics should have been used instead. This applies when the magnitudes of the stimuli can be only positive real, because then the measures of performance will be affected by the convexity bias. Unfortunately, it is not possible to simply compute the proposed geometric Weber fractions and geometric constant errors from the conventional arithmetic Weber fractions and arithmetic constant errors, because *W*_A_ and *CE*_A_ depend on both *CE*_G_ and *W*_G_ (lines in Figure 2C; prediction surfaces in Figures 4C,F). It will therefore be necessary to reanalyze the raw data. The size of the convexity bias will differ across studies because it depends on the width of the distribution, which will differ across conditions, tasks, and subjects.

## Author contributions

BF conceived of the study, performed the experiments, developed the novel measures, analyzed the data, and wrote the manuscript. PH conceived of the study, analyzed the data, and wrote the manuscript.

## Funding

This research is supported by the Deutsche Forschungsgemeinschaft (SFB/TRR 31 A6 to PH).

### Conflict of interest statement

The authors declare that the research was conducted in the absence of any commercial or financial relationships that could be construed as a potential conflict of interest.
