# Supplementary material for: Onset-Duration Matching of Acoustic Stimuli Revisited: Conventional Arithmetic vs. Proposed Geometric Measures of Accuracy and Precision
Source: Front Psychol. 2017 Jan 6;7:2013. doi: 10.3389/fpsyg.2016.02013 (PMC5216879; doi:10.3389/fpsyg.2016.02013)
Supplement: Supplementary file 1 [file Presentation1.pdf]

December 20, 2016

Supplementary Material

Onset-Duration Matching of Acoustic Stimuli Revisited: Conventional Arithmetic vs. Proposed Geometric Measures of Accuracy and Precision

Björn Friedrich & Peter Heil

Leibniz Institute for Neurobiology, Magdeburg, Germany

1 Derivation of the Prediction Function α_{pred}

Here, we derive the function α_{pred}, which predicts how the differences between the multiplicative normal (log-normal) and additive normal (normal) negative log-likelihoods change with the number of matches included in the fits, on the assumption that matches follow multiplicative normal distributions. Let

$$f_{\text{add}}\left(x \mid \mu_{\text{add}}, \sigma^2_{\text{add}}\right) := \frac{1}{\sqrt{2\pi\sigma^2_{\text{add}}}} \exp\left(-\frac{(x-\mu_{\text{add}})^2}{2\sigma^2_{\text{add}}}\right)$$

denote the probability density function of the additive normal distribution with parameters μ_{add} and σ^2_{add}, and let

$$f_{\text{mult}}\left(x \mid \mu_{\text{mult}}, \sigma^2_{\text{mult}}\right) := \frac{1}{\sqrt{2\pi\sigma^2_{\text{mult}}}\,x} \exp\left(-\frac{(\ln(x)-\ln(\mu_{\text{mult}}))^2}{2\sigma^2_{\text{mult}}}\right)$$

denote the probability density function of the multiplicative normal distribution with parameters μ_{mult} and σ^2_{mult}. The negative log-likelihoods of the additive normal and the multiplicative normal distributions are given by

$$\begin{aligned}
\ell_{\text{add}}\left(X \mid \mu_{\text{add}}, \sigma^2_{\text{add}}\right) &= -\sum_{k=1}^{n} \ln\left(f_{\text{add}}\left(x_k \mid \mu_{\text{add}}, \sigma^2_{\text{add}}\right)\right) \\
&= \frac{n}{2}\ln\left(2\pi\sigma^2_{\text{add}}\right) + \frac{n}{2} \\
&= \frac{n}{2}\left(\ln\left(2\pi\sigma^2_{\text{add}}\right) + 1\right)
\end{aligned}$$

and

$$\begin{aligned}
\ell_{\text{mult}}\left(X \mid \mu_{\text{mult}}, \sigma^2_{\text{mult}}\right) &= -\sum_{k=1}^{n} \ln\left(f_{\text{mult}}\left(x_k \mid \mu_{\text{mult}}, \sigma^2_{\text{mult}}\right)\right) \\
&= n\mu_{\text{mult}} + \frac{n}{2}\ln\left(2\pi\sigma^2_{\text{mult}}\right) + \frac{n}{2} \\
&= \frac{n}{2}\left(2\mu_{\text{mult}} + \ln\left(2\pi\sigma^2_{\text{mult}}\right) + 1\right) ,
\end{aligned}$$

respectively, and their difference is

$$\begin{aligned}
\Delta\ell\left(X \mid \mu_{\text{mult}}, \sigma^2_{\text{mult}}, \mu_{\text{add}}, \sigma^2_{\text{add}}\right) &= \ell_{\text{mult}}\left(X \mid \mu_{\text{mult}}, \sigma^2_{\text{mult}}\right) - \ell_{\text{add}}\left(X \mid \mu_{\text{add}}, \sigma^2_{\text{add}}\right) \\
&= \frac{n}{2}\left(2\mu_{\text{mult}} + \ln\left(2\pi\sigma^2_{\text{mult}}\right) + 1\right) - \frac{n}{2}\left(\ln\left(2\pi\sigma^2_{\text{add}}\right) + 1\right) \\
&= \frac{n}{2}\left(2\mu_{\text{mult}} + \ln\left(2\pi\sigma^2_{\text{mult}}\right) - \ln\left(2\pi\sigma^2_{\text{add}}\right)\right) \\
&= \frac{n}{2}\left(2\mu_{\text{mult}} + \ln\left(\frac{\sigma^2_{\text{mult}}}{\sigma^2_{\text{add}}}\right)\right) \\
&= \frac{n}{2}\ln\left(\frac{e^{2\mu_{\text{mult}}}\sigma^2_{\text{mult}}}{\sigma^2_{\text{add}}}\right) .
\end{aligned}$$

On the assumption that the matches follow a multiplicative normal distribution, the arithmetic variance (see Table 3) is given by $\mathrm{AV}(X) = \mathrm{e}^{2\mu_{\text{mult}}} \cdot \mathrm{e}^{\sigma^2_{\text{mult}}} \left(\mathrm{e}^{\sigma^2_{\text{mult}}} - 1\right)$. But $\mathrm{AV}(X)$ is also the best estimator for σ^2_{add}, so we can rewrite the difference between the negative log-likelihoods as a function of n and σ^2_{mult}:

$$\Delta\ell\left(n, \sigma^2_{\text{mult}}\right) = \frac{n}{2} \ln\left(\frac{\sigma^2_{\text{mult}}}{\mathrm{e}^{\sigma^2_{\text{mult}}}\left(\mathrm{e}^{\sigma^2_{\text{mult}}} - 1\right)}\right) = n \cdot \ln\left(\frac{\sigma_{\text{mult}}}{\sqrt{\mathrm{e}^{\sigma^2_{\text{mult}}}\left(\mathrm{e}^{\sigma^2_{\text{mult}}} - 1\right)}}\right) .$$

Finally, we substitute $W_{\text{G}} = \sigma_{\text{mult}}$, where W_{G} is the geometric Weber fraction derived in the results section of the main article, and take the derivative with respect to n to obtain the prediction function

$$\alpha_{\text{pred}}\left(W_{\text{G}}\right) = \frac{\partial \Delta\ell}{\partial n} = \ln\left(\frac{W_{\text{G}}}{\sqrt{\mathrm{e}^{W^2_{\text{G}}}\left(\mathrm{e}^{W^2_{\text{G}}} - 1\right)}}\right) .$$

2 Relationships between Arithmetic and Geometric Measures

Let X_{St} denote the distribution of matches to the standard stimulus with onset duration St. Here, we derive the relationships of the arithmetic constant error CE_{A} and Weber fraction W_{A} to the geometric constant error CE_{G} and Weber fraction W_{G} on the assumption that X_{St} follows a multiplicative normal distribution, i.e., $\ln\left(X_{St}\right) \sim \mathcal{N}\left(\ln(St) + CE_{\text{G}}, W^2_{\text{G}}\right)$. This allows us to use known relationships of arithmetic and geometric descriptive statistical measures to the parameters of a multiplicative normal distribution (Table 3). The arithmetic point of subjective equality, defined as $PSE_{\text{A}} \coloneqq \mathrm{AM}(X_{St})$, is given by

$$\begin{aligned} PSE_{\text{A}} &= \mathrm{e}^{\ln(St) + CE_{\text{G}} + \frac{1}{2}W^2_{\text{G}}} \\ &= St \cdot \mathrm{e}^{CE_{\text{G}} + \frac{1}{2}W^2_{\text{G}}} . \end{aligned}$$

The arithmetic constant error, defined as $CE_{\text{A}} \coloneqq \frac{PSE_{\text{A}} - St}{St}$, is therefore related to the geometric measures by

$$CE_{\text{A}} = \mathrm{e}^{CE_{\text{G}} + \frac{1}{2}W^2_{\text{G}}} - 1 .$$

Similarly, the arithmetic difference limen, defined as $DL_{\text{A}} \coloneqq \mathrm{AS}(X_{St})$, is given by

$$\begin{aligned} DL_{\text{A}} &= \mathrm{e}^{\ln(St) + CE_{\text{G}} + \frac{1}{2}W^2_{\text{G}}} \cdot \sqrt{\mathrm{e}^{W^2_{\text{G}}} - 1} \\ &= St \cdot \mathrm{e}^{CE_{\text{G}}}\sqrt{\mathrm{e}^{W^2_{\text{G}}}\left(\mathrm{e}^{W^2_{\text{G}}} - 1\right)} . \end{aligned}$$

The arithmetic Weber fraction, defined as $W_{\text{A}} \coloneqq \frac{DL_{\text{A}}}{St}$, is therefore related to the geometric measures by

$$W_{\text{A}} = \mathrm{e}^{CE_{\text{G}}}\sqrt{\mathrm{e}^{W^2_{\text{G}}}\left(\mathrm{e}^{W^2_{\text{G}}} - 1\right)} .$$

3 Tables

Table 1: Formulae to calculate arithmetic and geometric descriptive statistical measures.

**Statistical measure**	**Arithmetic**	**Geometric**
Mean	$\mathrm{AM}(X) = \frac{1}{n}\sum_{i=1}^{n} x_i$	$\mathrm{GM}(X) = \left(\prod_{i=1}^{n} x_i\right)^{\frac{1}{n}}$<br>$= \exp\big(\mathrm{AM}\left(\ln(X)\right)\big)$
Variance	$\mathrm{AV}(X) = \frac{1}{n}\sum_{i=1}^{n}\left(x_i - \mathrm{AM}(X)\right)^2$	$\mathrm{GV}(X) = \exp\left(\frac{1}{n}\sum_{i=1}^{n}\ln^2\left(\frac{x_i}{\mathrm{GM}(X)}\right)\right)$<br>$= \exp\big(\mathrm{AV}\left(\ln(X)\right)\big)$
Standard deviation	$\mathrm{AS}(X) = \sqrt{\mathrm{AV}(X)}$	$\mathrm{GS}(X) = \exp\left(\sqrt{\ln(\mathrm{GV}(X))}\right)$<br>$= \exp\big(\mathrm{AS}\left(\ln(X)\right)\big)$
Standard error	$\mathrm{ASE}(X) = \frac{1}{\sqrt{n}}\,\mathrm{AS}(X)$	$\mathrm{GSE}(X) = \mathrm{GS}(X)^{\frac{1}{\sqrt{n}}}$<br>$= \exp\big(\mathrm{ASE}\left(\ln(X)\right)\big)$

Table 2: Comparison of the additive Euclidean vector space $\mathbb{R}$ and the multiplicative Euclidean vector space $\mathbb{R}_{>0}$. Notice that x and y are arbitrary elements of the respective vector space and that $\lambda \in \mathbb{R}$.

**Operation/ Property**	$\mathbb{R}$	$\mathbb{R}_{>0}$
Vector addition	ordinary addition<br>$x + y$	ordinary multiplication<br>$x \cdot y$
Scalar multiplication	ordinary multiplication<br>$\lambda \cdot x$	ordinary exponentiation<br>x^λ
Scalar product	ordinary multiplication<br>$x \cdot y$	ordinary mult. of logarithms<br>$\ln(x) \cdot \ln(y)$
Scalar product norm	$\sqrt{x \cdot x} = \lvert x \rvert$	$\sqrt{\ln(x) \cdot \ln(x)} = \lvert \ln(x) \rvert$
Distance	$\lvert x - y \rvert$	$\lvert \ln(x) - \ln(y) \rvert$
Canonical probability dist.	additive normal<br>additive uniform	multiplicative normal<br>multiplicative uniform
Summary statistics	arithmetic<br>AM, AV, AS, ASE	geometric<br>GM, GV, GS, GSE

Table 3: Relationships of arithmetic and geometric descriptive statistical measures to the parameters of a multiplicative normal distribution of a random variable X, the logarithm of which is normally distributed, i.e., $\ln(X) \sim \mathcal{N}\left(\mu, \sigma^2\right)$.

**Statistical measure**	**Arithmetic**	**Geometric**
Mean	$\mathrm{AM}(X) = \mathrm{e}^{\mu + \frac{1}{2}\sigma^2}$	$\mathrm{GM}(X) = \mathrm{e}^{\mu}$
Variance	$\mathrm{AV}(X) = \mathrm{e}^{2\mu + \sigma^2}\left(\mathrm{e}^{\sigma^2} - 1\right)$	$\mathrm{GV}(X) = \mathrm{e}^{\sigma^2}$
Standard deviation	$\mathrm{AS}(X) = \mathrm{e}^{\mu + \frac{1}{2}\sigma^2}\sqrt{\mathrm{e}^{\sigma^2} - 1}$	$\mathrm{GS}(X) = \mathrm{e}^{\sigma}$
Standard error	$\mathrm{ASE}(X) = \mathrm{e}^{\mu + \frac{1}{2}\sigma^2}\sqrt{\left(\mathrm{e}^{\sigma^2} - 1\right)/n}$	$\mathrm{GSE}(X) = \mathrm{e}^{\sigma/\sqrt{n}}$
Median	$\mathrm{Med}(X) = \mathrm{e}^{\mu} = \mathrm{GM}(X)$	
Mode	$\mathrm{Mode}(X) = \mathrm{e}^{\mu - \sigma^2}$	
